# Comparative transcriptomic analysis provides insights into the development of a *Salvia splendens Ker-Gawler* mutant, *SX919M*

**DOI:** 10.1371/journal.pone.0213446

**Published:** 2019-03-14

**Authors:** Aixiang Dong, Jingjing Wang, Huali Zhang, Haibo Xin, Zhengnan Zhao, Fang Liang, Zijing Li, Richen Cong, Yan Lin, Lina Song, Lingling Tan, Pengwei Zhang, Rongfeng Cui

**Affiliations:** 1 Beijing Key Laboratory of Greening Plants Breeding, Beijing Institute of Landscape Architecture, Beijing, People’s Republic of China; 2 College of Biological Sciences and Biotechnology, Beijing Forestry University, Beijing, People’s Republic of China; 3 School of life sciences, Qingdao Agricultural University, Qingdao, Shandong, People’s Republic of China; 4 ShiJiaZhuang Academy of Agricultural and Forestry Sciences, Zhaoxian, Shijiazhuang, Hebei, People’s Republic of China; Dokuz Eylul Universitesi, TURKEY

## Abstract

*Salvia splendens* is a perennial, ornamental herbaceous flower that is widely cultivated as a bedding plant in gardens. The development of novel *S*. *splendens* cultivars and investigating the relevant molecular mechanisms are of great significance. In this study, RNA-sequencing and real-time PCR methods were used to analyze the possible molecular mechanism of *S*. *splendens* mutant, *SX919M*. From the wild-type *S*. *splendens 919CK*, we firstly selected a natural mutant, *SX919M*, which displayed multiple branches, clustered spheroids, and radial symmetrical inflorescence with higher numbers of calyces, ovules, stamens, and perianth tubes. Further, the RNA-seq was used to identify the differentially expressed genes (DEGs) in the mutant which included a total of 3568 upregulated and 3290 downregulated unigenes. We further observed that the indole alkaloid biosynthesis pathway showed the highest DEG enrichment, which was supported by a significant increase in the IAA content in mutant *SX919M*. In addition, we validated three DEGs, namely, CL2200.Contig2_All encoding methyl IAA esterase, CL12462.Contig1_All and CL12462.Contig2_All, which encoded strictosidine synthase, upregulated in mutant *SX919M*. We selected a novel *S*. *splendens* germplasm *SX919M* with a high ornamental value and determined that the upregulation of IAA biogenesis may be associated with its development.

## Introduction

*Salvia* L., with nearly 1000 species of shrubs, herbaceous perennials, and annuals, is the largest genus in the mint family (Lamiaceae: Nepetoideae: Mentheae: Salviinae) [1–4]. The genus is widely distributed throughout the world. Many species of this genus, such as *S*. *officinalis* and *S*. *miltiorrhiza* (Danshen), are extensively used for culinary purposes, essential oil production, and Chinese herbal remedies [1]. Additionally, species such as *S*. *splendens* are used as ornamental plants, valued for their flowers and aromatic foliage.

*S*. *splendens* (NCBI taxon ID: 180675), scarlet or tropical sage, is an herbaceous perennial species native to Brazil. Its life cycle is largely influenced by temperature. It is a perennial species in warmer regions and an annual one in cooler areas.

*S*. *splendens* is widely cultivated as a bedding plant in gardens all over the world because of its outstanding characteristics such as its dense flowers, dramatic colors (scarlet, purple, pink, blue, lavender, salmon, yellow green, white, and bicolor), and the long lasting flowering period (3–9 weeks or even longer) [2, 4]. In addition, it produces an insect-repellent fragrance, which enables it to grow stably without pests and diseases [1, 3].

Traditionally, a large number of new cultivars with different performances are obtained through a variety of hybridization and phenotypic selections. Novel cultivars are related to improvement in color, flowering time, life cycle, plant height, and/or moisture or temperature tolerance. However, the current understanding of the molecular mechanisms underlying such economically important characteristics of ornamental varieties is limited. To date, only few genetic markers are available for marker-assistant breeding or genetic modification [3]. Therefore, the strategy of RNA-Seq transcriptome analysis without the genome information should be the appropriate way to xplore the molecular mechanism of the *S*. *splendens* cultivars [5, 6].

In the current study, we selected *S*. *splendens* mutant, *SX919M*, from the traditional cultivar Qiji (*919CK*). *SX919M* is a natural mutation and exhibits multibranching and radiation symmetry inflorescence in ground load, which completely differs from *919CK*. The RNA-seq method was used to comparatively analyze differentially expressed genes, which provides hints in elucidating the molecular mechanism of *SX919M*. Changes in related hormone content validated the results of RNA-seq analysis. This study not only introduced an outstanding and valuable *S*. *splendens* natural variety *SX919M* that could be widely used in gardens, but also elucidated its molecular mechanism and identified related molecular markers.

## Results

### A *S*. *splendens Ker-Gawler* mutant, *SX919M*, confers dramatic changes in inflorescence

**Fig 1:**
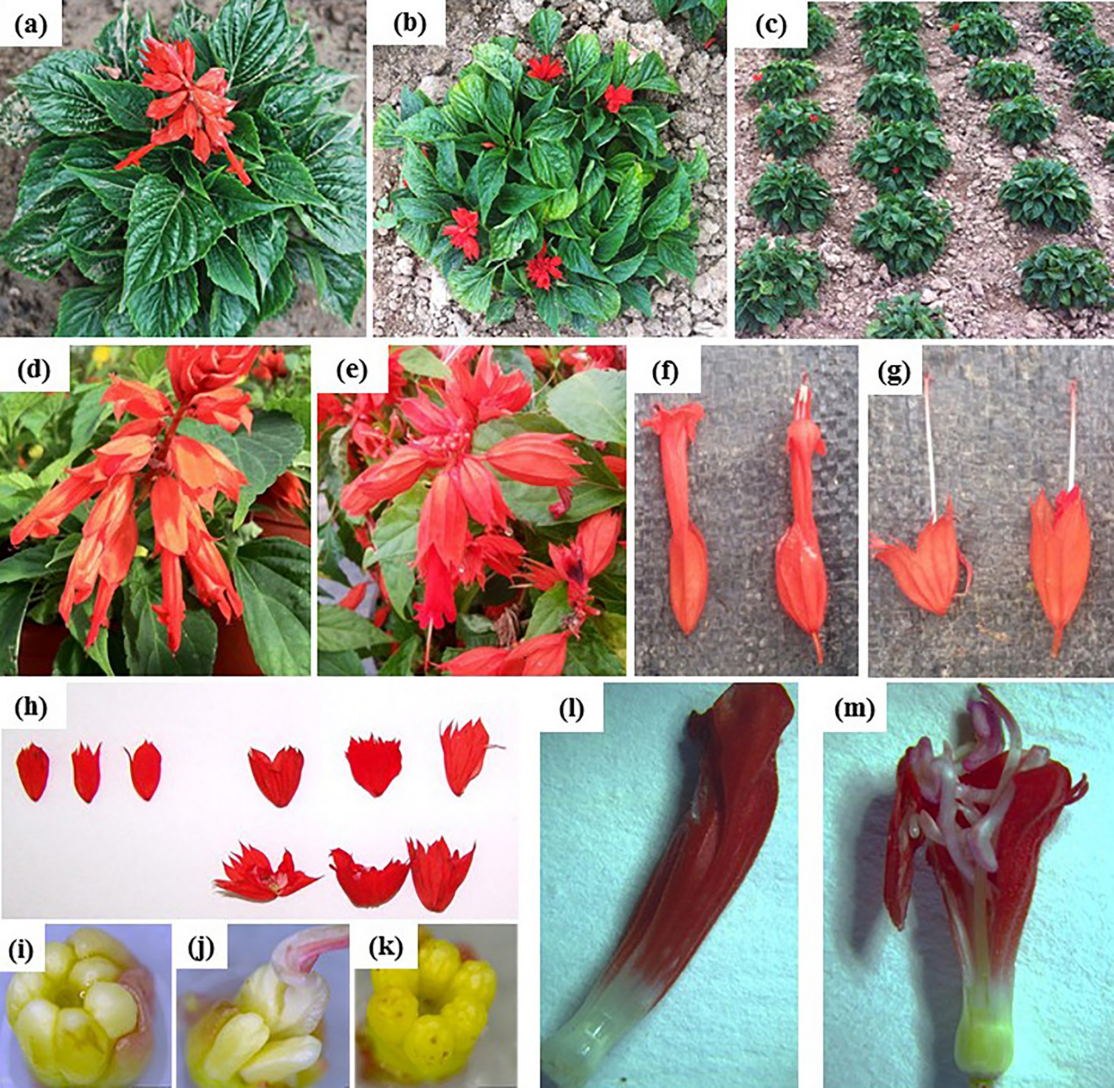
Phenotypic comparison of wild-type *Salvia splenden 919CK* and mutant *SX919M*. a and b, wild-type and mutant with multibranch and clustered spheroids; c, bedding population of the mutant; d and e, inflorescence of wild-type and mutant, respectively; f and g, flower organs of wild-type and mutant; h, sepals of wild-type (left) and mutant (right); i, stamen of wild-type; j and k, stamen of mutant; l and m, perianth tube of wild-type (left) and mutant (right).

*S*. *splenden* mutant *SX919M* is a natural mutant of the stable cultivar *S*. *splenden Ker-Gawler Qiji* (wild-type *919CK*), which was produced by the Beijing Institute of Landscape Architecture. Compared to wild-type (Fig 1A, 1D and 1F), the mutant has multiple branches and occur as clustered spheroids (Fig 1B and 1C). In addition, the inflorescence of mutant is abnormal, changing from bilateral to radial symmetry (Fig 1E), as well as a higher number of calyces, ovules, stamens and perianth tubes (Fig 1H, 1J, 1K, and 1M). Nevertheless, the abnormal inflorescence do not affect the fertilities of male and female lines of the asexual population of the mutant, which can produce mature seeds.

### RNA-seq analysis of wild-type *S*. *splendens Ker-Gawler 919CK* and mutant *SX919M*

To investigate the possible molecular mechanism of *S*. *splendens Ker-Gawler* mutant, RNA-seq analysis based on high-throughput sequencing was conducted to reveal changes in transcript abundance between the wild-type *S*. *splendens Ker-Gawler* and mutant. Three independent biological replicates of wild type and mutant were used in RNA-seq sequencing. Immature inflorescences and axillary buds were pooled from five individual plants, after which a total of six cDNA libraries were constructed.

The present study used a BGISEQ-500 sequencing platform to sequence the six libraries. The number of raw reads from each library ranged from 69.73 million to 72.22 million. After removing poor-quality reads, adaptor contamination, and low-quality regions, between 65.71 to 66.80 million clean reads were obtained from each library. Clean read rates is ranged from 92.5%-94.24% (S2 Table). After assembling all samples together and filtering the abundance using TIGCL software, we obtained 86,147 unigenes (S3 Table). In the all-unigene data, the number of unigenes with the lengths < 300 bp was 17,879 (20.75%), whereas those > 900 bp in length were 41,458 (48.12%).

The raw transcriptome data generated from this study were submitted to the Sequence Read Archive (SRA) of the National Center for Biotechnology Information (NCBI) as accession number SRP158242.

### GO annotation

The GO database is a collection of terms describing the biological functions of gene products in any organism. Generally, there were three independent categories of ontologies: biology process, cellular component, and molecular function [7]. To further analyze the function of the unigenes, the annotated information of the unigenes were obtained by aligning with seven functional databases. Finally, 45,557 (KEGG: 52.88%), 48,038 (KOG: 55.76%), 62,185 (NR: 72.18%), 41,797 (NT: 48.52%), 41,954 (Swiss-Prot: 48.70%), 17,095 (GO: 19.84%), and 51,735 (InterPro: 60.05%) unigenes were annotated (Table 1 and S4–S8 Tables).

**Table 1:** Annotation summary.

Annotation database	Annotated number	Percentage
Nr_annotation	62,185	72.18%
Nt_annotation	41,797	48.52%
Swissprot_annotation	41,954	48.70%
KEGG_annotation	45,557	52.88%
KOG_annotation	48,038	55.76%
Interpro_annotation	51,731	60.05%
GO_annotation	8,929	10.37%
All_annotation	86,147	100%

### DEGs and related pathway analysis

To investigate the possible molecular mechanism of the development of the mutant, we calculated the gene expression levels of wild-type and mutant and comparatively analyzed their expression levels. To validate the results of RNA-seq analysis, real-time PCR was conducted to detect the expression of nine unique genes that exhibited significant fold-changes when mutant was compared to wild-type.

**Fig 2:**
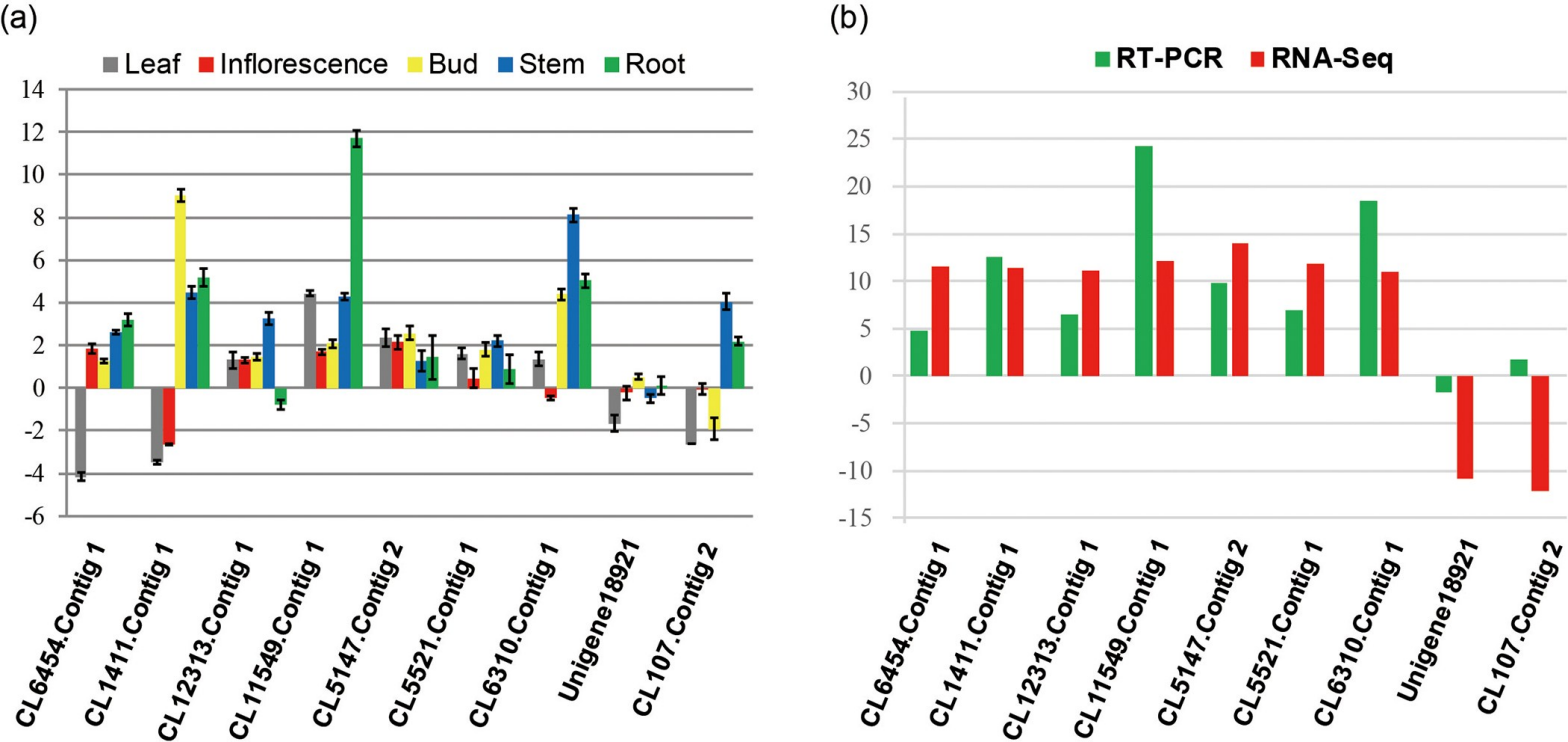
Validation of expression changes of DEGs between wild-type and mutant using real-time PCR analysis. a, Detection of changes in the expression of nine unigenes in various tissues using real-time PCR. b, Fold-change validation of the nine DEGs. The data were the average of three independent biological replicates. Unigene CL6454.Contig 1 encodes 3-hydroxyisobutyrate dehydrogenase-like 1 protein, CL1411.Contig 1 encodes NAC 29-like protein, CL12313.Contig 1 encodes acireductone dioxygenase, CL11549.Contig 1 encodes glutathione S-transferase T1-like protein, CL5147.Contig 2 encodes FAM133-like protein, CL5521.Contig 1 encodes light-induced protein, CL6310.Contig 1 encodes plasma membrane ATPase 4 protein, unigene18921 encodes hypothetical protein CDL12_16597, CL107.Contig 2 encodes copper chaperone.

Fig 2A shows that compared to wild-type, the nine genes in the mutant *SX919M* exhibited the different fold-changes in the leaves, inflorescence, buds, stems, and roots. When the total amount of transcripts was compared, the fold-changes coincided with those generated by RNA-seq analysis (Fig 2B). Of these, unigenes *CL6454*.*Contig1*, *CL1411*.*Contig1*, *CL12313*.*Contig1*, *CL11549*.*Contig1*, *CL5247*.*Contig2*, *CL5521*.*Contig1*, *CL6310*.*Contig1* were upregulated in mutant, whereas *Unigene18921* and *CL107*.*Contig2* were downregulated (Fig 2B, S9 and S10 Tables). The agreement between the real-time PCR and RNA-seq data reflects the reliability of RNA-seq analysis.

Analysis of DEGs has been shown to be an efficient approach in elucidating the molecular mechanism for the development of the mutant. Based on the identified unigenes, DEGs were further analyzed between the wild-type and mutant according to the criteria of fold-change ≥ 1 with an FDR<0.001 (S1 Fig, S9 and S10 Tables). Cluster analysis of DEGs suggested that the number of upregulated genes (3,568 unigenes) was generally higher than the downregulated genes (3,290 unigenes) when the wild-type was compared to mutant (S1 Fig). The majority (91.77%) of the unique genes (total of 76,425) had comparable expression levels between them.

**Fig 3:**
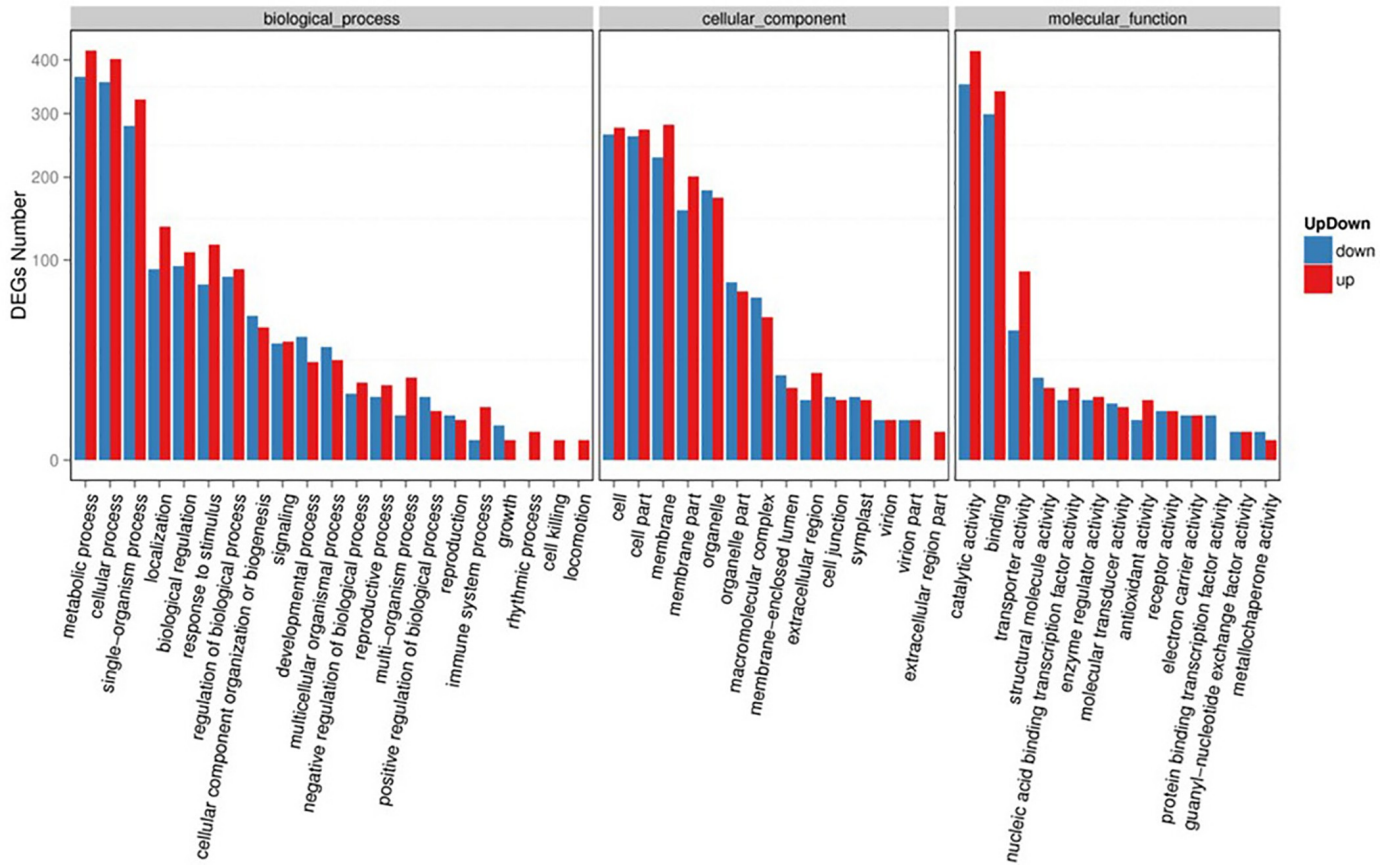
GO terms of DEGs.

For biology process ontology, proteins involved in metabolic, cellular, and single-organism processes were predominant. Under cellular component ontology, the GO terms cell, cell part, and membrane were mainly represented. For molecular function ontology, catalytic activity and binding were highly enriched with *S*. *splendens* unigenes (Fig 3). These distribution patterns indicate that *S*. *splendens* undergoes multiple developmental processes [7]. Furthermore, for the significantly enriched GO terms, there were more unigenes detected in mutant than in wild-type, which may indicate that more unigenes were needed to participate in various biological aspects, which in turn result in the observed phenotypic changes in *SX919M*.

**Fig 4:**
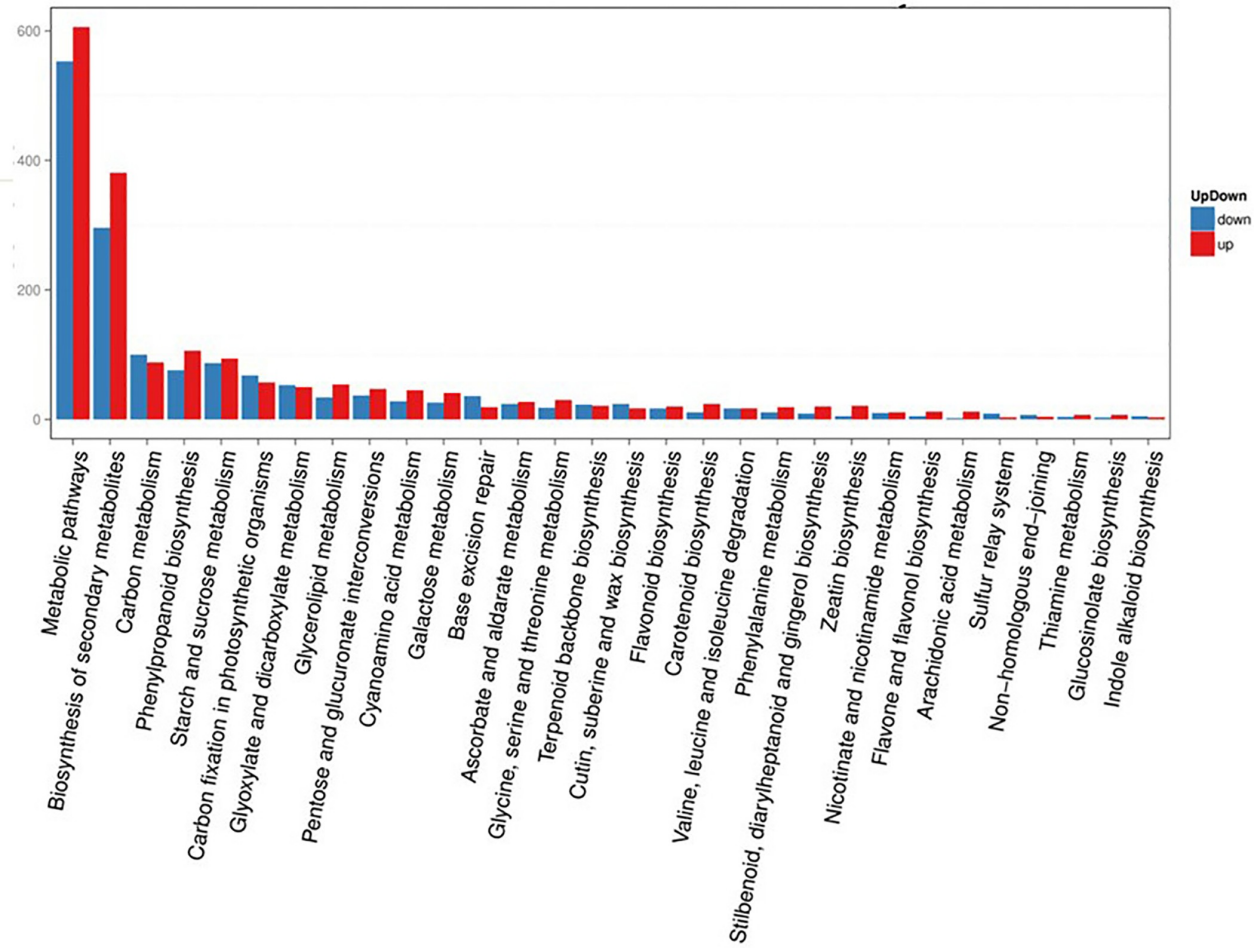
Analysis of DEG-enriched pathways. The X-axis represented the pathways, whereas the Y-axis shows the number of DEGs involved in the corresponding pathway.

With emphasis on biochemical pathways, KEGG pathway analysis can be used as an alternative way to categorize gene functions. These predicted pathways were generally involved in compound biosynthesis during growth and development such as degradation, utilization, assimilation, and pathways involved in the generation of precursor metabolites and energy. Enzymes encoded by the annotated unigenes were grouped into nearly all steps in several major plant metabolic pathways, including the carbon cycle, starch and sucrose, glyoxylate and dicarboxylate, glycerolipid, amino acids, galactose, ascorbate and aldarate, phenylalanine, nicotinate and nicotinamide, thiamine, and several important secondary metabolite biosynthesis pathways (Fig 4).

These results suggest that a large number of metabolic and biosynthesis activities occurred during the growth and development of *S*. *splendens*, especially during the development of the mutant. Among all of these pathways, “metabolic pathways” and “biosynthesis of secondary metabolism” were the most highly enriched (Fig 4), suggesting that DEGs involved in the two pathways may be related to the phenotypic changes in mutant. Moreover, in the two pathways, there were more upregulated genes than downregulated genes (Fig 4). Therefore, these DEGs may be associated with the mechanism of mutant *SX919M*, and their gene functions need to be further analyzed.

### The biosynthesis of indole-3-acetic acid (IAA) may be associated with the development of *S*. *splendens* mutant, *SX919M*

**Fig 5:**
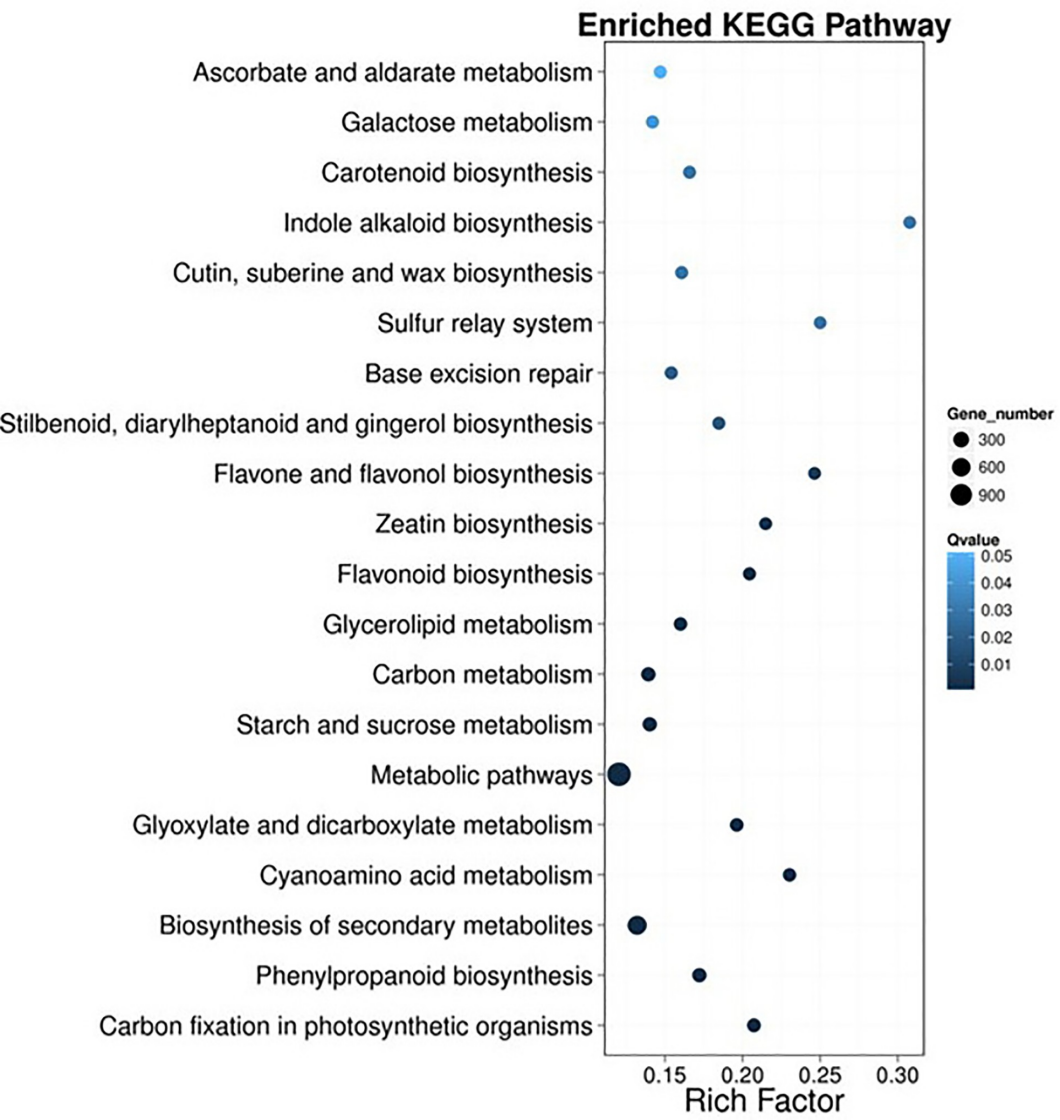
Pathway enrichment analysis of DEGs by bubble picture. The X-axis represents the enrichment factor, whereas the Y-axis represents the name of the pathway. The color indicates the Q-value (high: white, low: blue); a lower Q-value indicates greater enrichment. Point size indicates the number of DEGs (the bigger dots refer to larger amounts). Rich factor refers to the level of enrichment, which is the quotient of the foreground value (the number of DEGs) and background value (total gene amount). The larger the value, the more significant the enrichment.

To elucidate the mechanism of *SX919M* development, we further investigated the enriched KEGG pathways. Fig 5 showed that the “indole alkaloid biosynthesis” pathway showed the highest DEG enrichment, suggesting the importance of this process in the development of the mutant. To validate whether the indole alkaloid biosynthesis pathway is associated with the development of mutant, we compared the levels of various hormones such as Indol-3-acetic acid (IAA), ribosylzeatin (ZR) and gibberellin (GA3) (Table 2).

**Table 2:** Content comparisons of IAA, ZR and GA3 between the wild type and the mutant.

Sample	IAA (ng/g)	ZR (ng/g)	GA3 (ng/g)
Wild-type *SX919M*	2.92±0.23 **	4.45±0.33	0.19±0.02
Mutant *919CK*	1.63±0.28	4.87±0.15	0.15±0.02

Note: The values (mean±*SD*) are the average of three independent replicates. The significances of T-test by 2-tailed method were indicated by asterisks

** designate significance at P < 0.001.

The results showed that the IAA content of mutant (2.92 ± 0.23 ng/g) was markedly higher than that of wild-type (1.63 ± 0.28 ng/g), whereas the contents of ZR and GA3 were comparable between them (ZR, 4.45 ± 0.33 ng/g and 4.87 ± 0.15 ng/g; GA3, 0.19 ± 0.02 ng/g and 0.15 ± 0.02 ng/g, respectively). Combined with DEG enrichment in “indole alkaloid biosynthesis”, the observed higher IAA content reflected that the phenotypic changes in mutant may be relevant to the increase in IAA content.

### Key genes responsible for *S*. *splendens Ker-Gawler* mutant, *SX919M*

**Fig 6:**
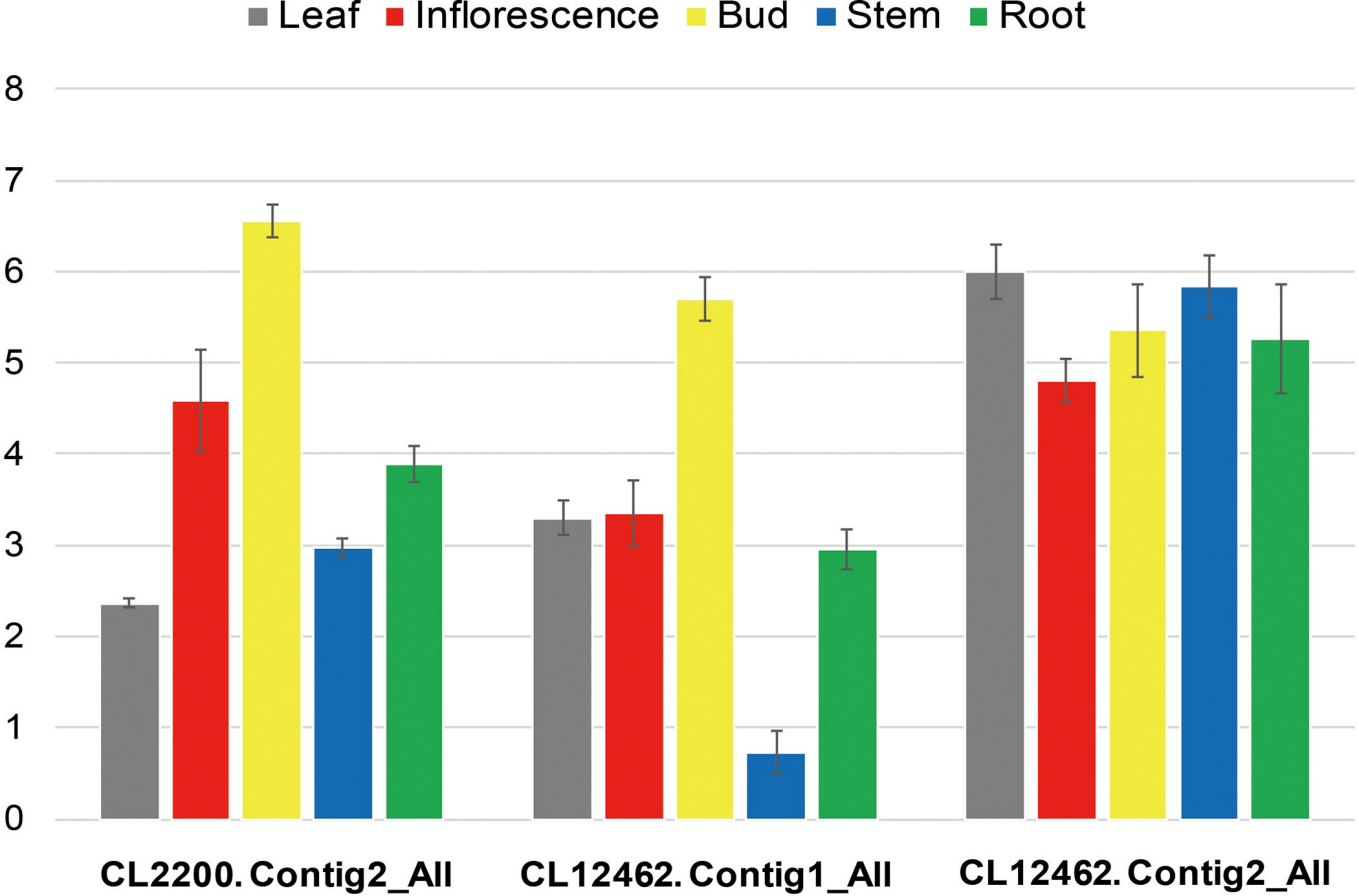
Validation of expression of unigenes that are related to indole alkaloid biosynthesis by real-time PCR. Unigene CL2200.Contig2_All encodes methyl esterase 3 protein, CL12462.Contig1_All encodes alkaloid synthase, and CL12462.Contig2_All encodes strictosidine synthase-like 6.

Through DEG analysis, three unique genes (CL12462. Contig1_All, Contig2_All, and CL2200. Contig2_All) involving the “indole alkaloid biosynthesis” pathway were obtained (Table 3). First, we detected the expression profiles using real-time PCR to validate the results of RNA-seq. Fig 6 shows that compared to the wild-type, three of these were upregulated in the mutant to different degrees. For CL12462.Contig1_All and CL2200.Contig2_All, the expression signal in the reproductive organs (inflorescence and bud) were significantly higher than that in the leaves, stems, and roots, whereas for the CL12462.Contig2_All, the examined tissues presented similar levels of transcription (Fig 6). GO analysis indicated that the ortholog of CL2200.Contig2_All was AT2G23610.2, which encodes a protein with methyl IAA esterase activity and functioned mainly in the stage of flowering and petal differentiation and expansion [8]. CL2200.Contig2_All was upregulated in mutant, which possibly catalyzes IAA biosynthesis. Moreover, the significant transcript accumulation of CL2200.Contig2_All in inflorescence and buds may partially explain the observed phenotypic changes in mutant. In addition, CL12462.Contig1_All was the ortholog of At3g51450 in *Arabidopsis*, which encodes a alkaloid biosynthesis-related protein with strictosidine synthase activity [9]. In rice, a similar ortholog TGW6 can convert IAA-glucose into free IAA [10]. Therefore, CL12462.Contig1_All and CL12462.Contig2_All may play important roles in IAA biosynthesis in mutant.

**Table 3:** Candidate DEGs involved in the indole alkaloid biosynthesis.

Unigene	Length	*919CK*-Expression	*SX919M*-Expression	log2FoldChange (*SX919M*/*919CK*)	FDR	P-value
*CL12462*.*Contig1_All*	1287	2.93	6.14	1.07	1.82929E-05	2.16968E-06
*CL12462*.*Contig2_All*	1383	0.24	7.59	4.98	7.21201E-39	9.66416E-41
*CL2200*.*Contig2_All*	909	1.95	5.25	1.43	2.48795E-05	3.02618E-06

## Discussion

In this study, we selected an outstanding *S*. *splenden* mutant, *SX919M*, from a traditional *S*. *splenden* cultivar *Ker-Gawler* Qiji (wild-type *919CK*). When compared with the wild-type, *SX919M* is characterized with multiple branches, clustered spheroids, and radial symmetrical inflorescence (Fig 1). Generally, *SX919M* is a valuable germplasm for garden landscaping of public parks. To investigate the possible molecular mechanism of its development, we performed a comparative transcriptome analysis between wild-type and mutant.

### Enriched pathways in the development of *S*. *splendens*

Through GO term analysis, we predicted the unigene-encoding proteins in three functional categories, namely, biological process, cellular component, and molecular function. In the category of biological process, metabolic, cellular, and single-organism processes were predominant, as indicated by DEG enrichment (Fig 4). In 2014, two different types of branching *S*. *splendens* materials (Strain 35 and Cailinghong) were also analyzed by transcriptome sequencing [3]. GO term analysis conducted by the two studies showed similar most-enriched pathways (catalytic activity and binding activity) for the category of molecular function and cell-related pathways for the category of cellular component (Fig 3) [3]. However, physiological process, development, and cellular process were the top three subcategories in the functional category of biological process between Strain 35 and Cailinghong, which differed from the results between *919CK* and mutant *SX919M* because metabolic process was the one of the three enriched processes in the category of biological process (Fig 3). The difference may be attributable to phenotypic variations observed in the two studies. In our study, the obvious phenotypic difference between wild-type *919CK* and mutant *SX919M* was not only the multiple branches in mutant, but also the significant inflorescence changes from bilateral to radial symmetry with clustered spheroids. In addition, the major phenotypic difference between Strain 35 and Cailinghong was the branching type. It is thus seen that the DEG enrichment of the functional category of metabolic process may be related to the phenotypic difference from wild-type *919CK*.

### The role of IAA in plant development

Indole-3-acid is a plant hormone involved in various aspects of plant growth and development, such as embryogenesis, vascular differentiation, fruit set and development, and senescence [11–13]. The amount of active IAA in specific tissues is tightly regulated and determined by an array of metabolic processes, including the regulation of its synthesis, transport to or from specific cells or tissues, IAA inactivation and reactivation, and degradation via multiple oxidative pathways [7]. In this study, we first used the RNA-seq strategy to investigate the possible molecular mechanism of the development of *S*. *splendens Ker-Gawler* mutant, *SX919M*. Through the GO term and KEGG enrichment analyses, we found that a majority of DEGs are involved in metabolic pathways and the biosynthesis of secondary metabolism, especially, indole alkaloid biosynthesis showed the highest DEG enrichment (Figs 4 and 5). This result was further supported by the significant increase in the plant hormone IAA in mutant (Table 2) and the upregulation of three important DEGs, *CL12462*.*Contig1_All*, *CL12462*.*Contig2_All*, and *CL2200*.*Contig2_All* (Fig 6 and Table 3). The ortholog of *CL2200*.*Contig2_All* is *AT2G23610*.*2* in *Arabidopsis*, which encodes MeIAA esterase [14]. Generally, the *Arabidopsis* esterase family gene *AtMES* is involved in the biosynthesis of IAA, which can convert inactive methyl indole-3-acetic acid into its free form by hydrolysis [8]. Previous studies have shown that IAA methylation is essential to maintain the specific concentration range in the endodermis that perceives gravity and functions in the elongation of the primary root [9, 14]. Similarly, tobacco (*Nicotiana tabacum*) protein SABP2 (salicylic acid binding protein 2) hydrolyzes methyl salicylate (MeSA) to salicylic acid (SA), which activates the systemic acquired resistance [10]. In addition, another DEG, *CL12462*.*Contig1_All*, is also upregulated in mutant, which is the counterpart of *At3g51450* in *Arabidopsis*, encoding an alkaloid biosynthesis-related protein with strictosidine synthase activity [15]. In rice, strictosidine synthase TGW6 catalyzes IAA-glucose into free IAA, which regulates grain development [16]. In the study, a comparison between wild-type *S*. *splendens* and mutant revealed multibranch and radiation-symmetric inflorescence and higher IAA content, which is in compliance with the results of a previous research which reported that blocking miR396 greatly increases grain yield by modulating the development of auxiliary branches and spikelets through the direct induction of growth regulating factor 6 (*OsGRF6*) gene. The upregulation of *OsGRF6* results in the coordinated activation of auxin (IAA) biosynthesis, auxin response factors, and branch and spikelet development-related transcription factors [17].

In the study, the strategy of RNA-seq has shown that the increase in IAA levels and the overexpression of key genes in the IAA biosynthesis pathways are apparently related to the development of the *S*. *splendens Ker-Gawler* mutant, *SX919M*. Nevertheless, more molecular biological experiments are required to be conducted to further demonstrate the specific molecular mechanism underlying the establishment of mutant *SM919M*.

## Materials and methods

### Plant materials

*S*. *splendens*, a member of genus *Salvia*, family *Lamiaceae*, with colorful flowers and long ornamental period, has been extensively used in Chinese garden afforestation from 1980’s. *S*. *splenden Ker-Gawler Qiji* (wild-type *919CK*) is a traditional cultivar, which has no branch with fresh green leaves, red calyx and corolla (Fig 1A). It is an important flower for Chinese festivals due to its high ornamental value. The mutant, *SX919M*, was found in the population of *S*. *splenden Ker-Gawler* cultivar *Qiji* (*919CK*) under the normal culturing condition in flower nursery of Beijing Institute of Landscape Architecture. It was produced naturally without treated by physical or chemical methods. The novel phenotypes of mutant made it more popular in Chinese garden afforestation and its molecular bases for the development of mutant need to be discovered.

### RNA extraction and library construction

The immature inflorescence and axillary buds of five wild-type *S*. *splendens Ker-Gawler 919CK* and mutant *SX919M* lines were separately pooled as one sample replicate. Three replicates for the wild-type and mutant were prepared for total RNA extraction. Total RNA was isolated using an RNeasy plant Mini (Qiagen, Duesseldorf, Germany) according to the manufacturer’s instructions. DNA contamination in the total RNA was removed using DN*a*se I (Takara, Shiga, Japan). The purity, concentration, and integrity of the total RNA were assessed using a NanoPHotometer spectrophotometer (IMPLEN, München, Germany), a Qubit RNA assay kit with a Qubit 2.0 Fluorometer (Thermo Fisher Scientific, Massachusetts, America), and an RNA Nano 6000 assay kit with a Bioanalyzer 2100 system (Agilent Technologies, California, America). Sequencing libraries were generated using an NEBNext UltraRNA Library Prep Kit for Illumina (NEB, Massachusetts, America) following the manufacturer’s recommendations. Briefly, mRNA was purified using polyT oligo-attached magnetic beads. After fragmentation, the cDNA was synthesized, and NEBNext Adaptors with hairpin loop structures were ligated and used in hybridization. PCR products were purified (AMPure XP system, California, America), and library quality was assessed on an Agilent Bioanalyzer 2100 system. Finally, six libraries were successfully constructed.

### Transcriptome analysis

The libraries were deep sequenced using a BGISEQ-500 sequencing platform. To obtain the clean data for further analysis, the raw data were screened, removing reads with adaptors, poly-N homopolymers, and those of low-quality.

For sequencing projects without a good reference genome, clean reads have to be assembled after sequencing to obtain a reference sequence that could be used in the subsequent analysis. After filtering the reads, we used Trinity to perform *de novo* assembly with the remaining clean reads [18]. TGICL was used on cluster transcripts to remove abundance and obtain unigenes [19]. Then, the unigenes were got after assembling all samples together and filtering the abundance followed by the calculation of the total length, average length, N50, and GC content of the unigenes.

The unigenes were annotated by aligning with the non-redundant protein sequence database (NR), nucleotide sequence database (NT), Swiss-Prot database, Kyoto Encyclopedia of Genes and Genomes (KEGG) database, Gene Ontology (GO) database, and protein sequence analysis & classification (InterPro) database. Furthermore, TransDecoder was used to detect the coding sequence (CDS).

The unigenes that aligned to the NR database were annotated to the GO database using Blast2GO [20], and their distribution in the three functional categories, namely, biological process, cellular component, and molecular functions was determined.

We used TransDecoder software to identify the candidate coding region in each unigenes. The longest open reading frame (ORF) was selected and was used as query in a BLAST search with Swiss-Prot and Hmmscan database to detect Pfam protein homology sequences to predict the CDS. After assembly, we predicted unigenes that encode plant transcription factor (TFs).

### Gene expression and DEG analysis

Based on the resulting assembly, we mapped all the clean reads of each sample to the unigenes using Bowtie2 software [21] and calculated the gene expression level with RSEM [22].

Box-plot was adopted to demonstrate the distribution of gene expression level. A gene expression density figure described changes in gene abundance. We also calculated the gene transcript abundance in different FPKM intervals.

Based on the gene expression level, we obtained the differentially expressed genes (DEGs) between the wild-type and mutant. We used DEGseq, DEseq2, EBseq, NOIseq, and PossionDis algorithms to detect the DEGs. Then, we performed GO classification and DEG functional enrichment. GO analysis showed that the DEGs enriched the following categories, namely, molecular biological function, cellular component, and biological process. We then performed KEGG pathway classification and functional enrichment using the DEGs. GO terms with corrected P values < 0.05 were considered significantly enriched.

### Quantitative real-time PCR

Leaves, flowers, buds, stems, and roots were collected from the wild-type *919CK* and mutant *SX919M*, respectively. All samples were flash frozen in liquid nitrogen. Total RNA was isolated using an E.Z.N.A. RNA probe purification kit (Omega bio-tek, Georgia, America). The concentration and quality of 10 RNA samples were determined using a NanoDrop 2000 spectrophotometer. First-strand cDNA was synthesized using a PrimeScript RT reagent kit with a gDNA Eraser kit (Takara, Shiga, Japan) following the manufacturer’s protocol. qRT-PCR amplification was conducted using a 7500 Real Time PCR System with TB Green *Premix Ex Taq* II (Takara, Shiga, Japan). The 20-μL reaction mixture included 1 μL of cDNA, 10 μL TB Green *Premix Ex Taq* II (Tli RNaseH plus), 0.4 μL 50×ROX Reference Dye, and 0.6 μL each of a 10-μM forward and reverse primer. The PCR conditions were as follows: pre-denaturation of 95°C for 10 s; followed by 45 cycles at 95°C for 5 s, 60°C for 30 s, and 72°C for 30 s. The information on the primers is listed in S1 Table. The final data were the average values of three independent biological replicates.

### Hormone detection

Hormone levels were determined by Zoonbio Biotechnology Co., Ltd. (Nanjing, China) using HPLC-MS/MS. Indol-3-acetic acid (IAA), ribosylzeatin (ZR) and gibberellin (GA3) were extracted and purified using 1 g of frozen fresh samples. Then, the purified products were subjected to HPLC-MS/MS analysis. HPLC (Agilent 1260) analysis was conducted using a ZORBAX SB-C18 column (2.1 mm×150 mm; 3.5 μm) (Agilent Technologies, California, America) at 30°C. The mobile phase A solvent consisted of methanol/0.1% formic acid, and the mobile phase B solvent consisted of water/0.1% methanoic acid. The input volume was 2 μL. The parameters for MS analysis were set as follows: spray voltage, 4,500 V; pressure of the air curtain, nebulizer, and aux gas were 15 psi, 65 psi, and 70 psi, respectively; and atomizing temperature, 400°C. Measurements were obtained for three biological replicates of each sample. The results were analyzed using Microsoft Excel.

## Supporting information

S1 Fig Fold-change analysis of DEGs by volcano plot. Red dots represent the 3568 upregulated unigenes (log_2_(fold-change) ≥ 1, FDR≦0.001) and the blue dots are the 3290 downregulated unigenes (log_2_(fold-change)≦-1, FDR≦0.001). Gray dots are not DEGs (absolute value of log_2_(fold-change) <1, FDR>0.001). The X-axis is the fold-change in the expression after log_2_ transformation and the Y-axis represents the significance after the -log_10_ transformation.
(JPG)

S1 Table The primer set used in this study. (XLSX)

S2 Table Clean reads quality metrics. (XLSX)

S3 Table Quality metrics of Unigenes. (XLSX)

S4 Table All unigenes annotated by KEGG database in the study. (XLSX)

S5 Table All unigenes annotated by KOG database in the study. (XLSX)

S6 Table All unigenes annotated by NR database in the study. (XLSX)

S7 Table All unigenes annotated by NT database in the study. (XLSX)

S8 Table All unigenes annotated by SWISSPROT database in the study. (XLSX)

S9 Table Gene expression was up-regulated in DEGs. (XLSX)

S10 Table Gene expression was down-regulated in DEGs. (XLSX)
