# Supplementary figures and images for: Comparative transcriptomic analysis provides insights into the development of a Salvia splendens Ker-Gawler mutant, SX919M
Source: PLoS One. 2019 Mar 14;14(3):e0213446. doi: 10.1371/journal.pone.0213446 (PMC6417697; doi:10.1371/journal.pone.0213446)

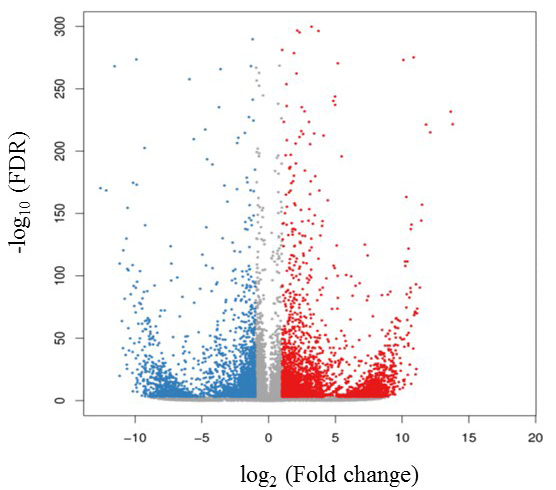

Supplement: S1 Fig — Red dots represent the 3568 upregulated unigenes (log2(fold-change) ≥ 1, FDR≦0.001) and the blue dots are the 3290 downregulated unigenes (log2(fold-change)≦-1, FDR≦0.001). Gray dots are not DEGs (absolute value of log2(fold-change) <1, FDR>0.001). The X-axis is the fold-change in the expression after log2 transformation and the Y-axis represents the significance after the -log10 transformation. (JPG) [file pone.0213446.s001.jpg]
